# Annealing Effect on the Characteristics of Co_40_Fe_40_W_10_B_10_ Thin Films on Si(100) Substrate

**DOI:** 10.3390/ma14206017

**Published:** 2021-10-13

**Authors:** Wen-Jen Liu, Yung-Huang Chang, Yuan-Tsung Chen, Chun-Yu Chang, Jian-Xin Lai, Shih-Hung Lin, Te-Ho Wu, Po-Wei Chi

**Affiliations:** 1 Department of Materials Science and Engineering, I-Shou University, Kaohsiung 84001, Taiwan; jurgen@isu.edu.tw; 2 Bachelor Program in Interdisciplinary Studies, National Yunlin University of Science and Technology, 123 University Road, Section 3, Douliou 64002, Yunlin, Taiwan; changyhu@yuntech.edu.tw; 3 Graduate School of Materials Science, National Yunlin University of Science and Technology, 123 University Road, Section 3, Douliou 64002, Yunlin, Taiwan; M10847004@yuntech.edu.tw (C.-Y.C.); M11047007@yuntech.edu.tw (J.-X.L.); wuth@yuntech.edu.tw (T.-H.W.); 4 Department of Electronic Engineering, National Yunlin University of Science and Technology, 123 University Road, Section 3, Douliou 64002, Yunlin, Taiwan; isshokenmei@yuntech.edu.tw; 5 Institute of Physics, Academia Sinica, Nankang, Taipei 11529, Taiwan; jacky01234567891@hotmail.com

**Keywords:** annealed Co_40_Fe_40_W_10_B_10_ thin films, magnetocrystalline anisotropy, low-frequency alternating current magnetic susceptibility (χ_ac_), optimal resonance frequency (f_res_), surface energy

## Abstract

This research explores the behavior of Co_40_Fe_40_W_10_B_10_ when it is sputtered onto Si(100) substrates with a thickness (t_f_) ranging from 10 nm to 100 nm, and then altered by an annealing process at temperatures of 200 °C, 250 °C, 300 °C, and 350 °C, respectively. The crystal structure and grain size of Co_40_Fe_40_W_10_B_10_ films with different thicknesses and annealing temperatures are observed and estimated by an X-ray diffractometer pattern (XRD) and full-width at half maximum (FWHM). The XRD of annealing Co_40_Fe_40_W_10_B_10_ films at 200 °C exhibited an amorphous status due to insufficient heating drive force. Moreover, the thicknesses and annealing temperatures of body-centered cubic (BCC) CoFe (110) peaks were detected when annealing at 250 °C with thicknesses ranging from 80 nm to 100 nm, annealing at 300 °C with thicknesses ranging from 50 nm to 100 nm, and annealing at 350 °C with thicknesses ranging from 10 nm to 100 nm. The FWHM of CoFe (110) decreased and the grain size increased when the thickness and annealing temperature increased. The CoFe (110) peak revealed magnetocrystalline anisotropy, which was related to strong low-frequency alternative-current magnetic susceptibility (χ_ac_) and induced an increasing trend in saturation magnetization (Ms) as the thickness and annealing temperature increased. The contact angles of all Co_40_Fe_40_W_10_B_10_ films were less than 90°, indicating the hydrophilic nature of Co_40_Fe_40_W_10_B_10_ films. Furthermore, the surface energy of Co_40_Fe_40_W_10_B_10_ presented an increased trend as the thickness and annealing temperature increased. According to the results, the optimal conditions are a thickness of 100 nm and an annealing temperature of 350 °C, owing to high χ_ac_, large Ms, and strong adhesion; this indicates that annealing Co_40_Fe_40_W_10_B_10_ at 350 °C and with a thickness of 100 nm exhibits good thermal stability and can become a free or pinned layer in a magnetic tunneling junction (MTJ) application.

## 1. Introduction

Ever since the conception of nanocrystalline materials by Rozlin in 2012, the CoFe alloy found in magnetic materials has presented excellent soft magnetic properties, categorized by high saturation magnetization (Ms) and a high Curie temperature (T_C_) [1]. This topic has continued to attract the attention of scholars researching CoFe material, which can be applied to magnetic equipment for sensor, actuator, and read-write recorder applications [2,3,4,5,6]. The CoFe matrix with magnetic properties combines with boron (B) to form CoFeB material, which is a soft magnetic material that is applied widely to spin electronic devices. The thickness of the CoFeB thin film is thin enough that it shows perpendicular magnetic anisotropy (PMA) and is applied to magnetoresistance random access memory (MRAM). CoFeB can be a free layer or pinned layer for increasing ferromagnetic (FM)/antiferromagnetic (AFM) exchange-biasing anisotropy in magnetic tunnel junction (MTJ), creating large tunnel magnetoresistance (TMR) [7,8,9,10,11,12]. Among all metals, tungsten (W) has a high melting point, high tensile strength, and thermal conductivity, as well as a low thermal expansion coefficient. The addition of refractory elements such as W to the CoFeB system is worthwhile to study their specific properties. In 2012, Pai et al. examined the effect of W as a seed layer on MTJ and observed different crystal structures formed by single-layer W films due to a different thickness and a spin Hall angle [13]. An experiment in 2015 found that the W layer influenced the PMA effect at different annealing temperatures [14]. The PMA has high stable efficiency at high temperatures with the CoFeB alloy presenting the major effect of the PMA simultaneously, indicating that the W layer probably has the factor of heat resistance and is realized at a higher annealing temperature in order to obtain a strong PMA feature. In 2016, researchers studied the electrodeposition efficiency of CoFeW films in a citrate solution by changing the chemical composition ratio of the films with different W content in the electrolytes. The morphology, microstructure, and magnetic properties of the films were analyzed. A CoFeW alloy with low coercivity (H_C_) and high saturation magnetization (M_S_) was obtained [15]. Findings show that the coating of the CoFeW alloy with a high PH value produces better magnetic properties. However, the coating of the CoFeW alloy with a low PH value causes a lack of surface tension and the phenomenon of the grain size to decrease. Adding W into the CoFe alloy can increase its hardness, durability, corrosion resistance, and heat resistance [16]. However, excessive B content leads to a decrease in saturation magnetization at high temperatures [17]. Higher saturation magnetization has the advantage of heating stability. Due to the above reasons, magnetic components are usually operated in a higher temperature environment than room temperature (RT). CoFeWB film is usually inserted into MTJ as a free layer, pinned layer, or combined with other layers in a multilayer structure. It can be widely used in magnetic and semiconductor applications. Adhesion is an important factor in CoFeWB film. The experiment investigates Co_40_Fe_40_W_10_B_10_ films deposited on Si (100) substrates when an annealing process occurs at 200 °C, 250 °C, 300 °C, and 350 °C, respectively. This experiment elected to add B and W into CoFe material and investigated their specific properties, including structure, adhesion, and magnetic characteristics, after annealing treatments.

## 2. Materials and Methods

Co_40_Fe_40_W_10_B_10_ with a thickness of 10–100 nm was sputtered onto Si(100) substrate at room temperature (RT) by a magnetron DC sputtering direct method of 50 W power and under the following four conditions: (a) annealed at a treatment temperature (T_A_) of 200 °C for 1 h, (b) annealed at 250 °C for 1 h, (c) annealed at 300 °C for 1 h, and (d) annealed at 350 °C for 1 h. The chamber base pressure was 8.5 × 10^−7^ Torr, and the Ar working pressure was 3 × 10^−3^ Torr. The pressure in the ex-situ annealed condition was 3 × 10^−3^ Torr with a selected Ar gas. The target alloy composition of CoFeWB was Co (40%), Fe (40%), W (10%), and B (10%). The structure of the CoFeWB thin films was detected by grazing incidence X-ray diffraction (XRD) patterns obtained with CuKα1 (PAN analytical X’pert PRO MRD, Malvern Panalytical Ltd, Cambridge, UK) and a low angle diffraction incidence of roughly 2 degrees. The in-plane low-frequency alternate-current magnetic susceptibility (χ_ac_) and hysteresis loop of the Co_40_Fe_40_W_20_ were studied using an χ_ac_ analyzer (XacQuan, MagQu Co. Ltd. New Tapei City, Taiwan) and an alternating gradient magnetometer (AGM, PMC, Westerville, OH, USA). Moreover, in χ_ac_ measurement, the χ_ac_ analyzer was used to calibrate the standard sample under the action of an external magnetic field. Then, the sample was inserted into the χ_ac_ analyzer. The driving frequency was between 10 and 25,000 Hz. χ_ac_ was measured using magnetization. All test samples had an equivalent shape and size to eliminate demagnetization. The χ_ac_ valve acted as an arbitrary unit (a.u.) because the AC result corresponded to the reference standard sample and could have been a comparison value. The connection between magnetic susceptibility and frequency was measured by an χ_ac_ analyzer. The best resonance frequency (f_res_) was measured by an χ_ac_ analyzer and represented the frequency of the maximum χ_ac_. Before measurement, the contact angle was properly air cleaned on the surface. The contact angle of the CoFeWB film was measured with deionized (DI) water and glycerol. The contact angle was measured when the samples were taken out of the chamber. The surface energy was obtained by measuring the contact angle and employing specific calculations [18,19,20].

## 3. Results

### 3.1. X-ray Diffraction

**Figure 1:**
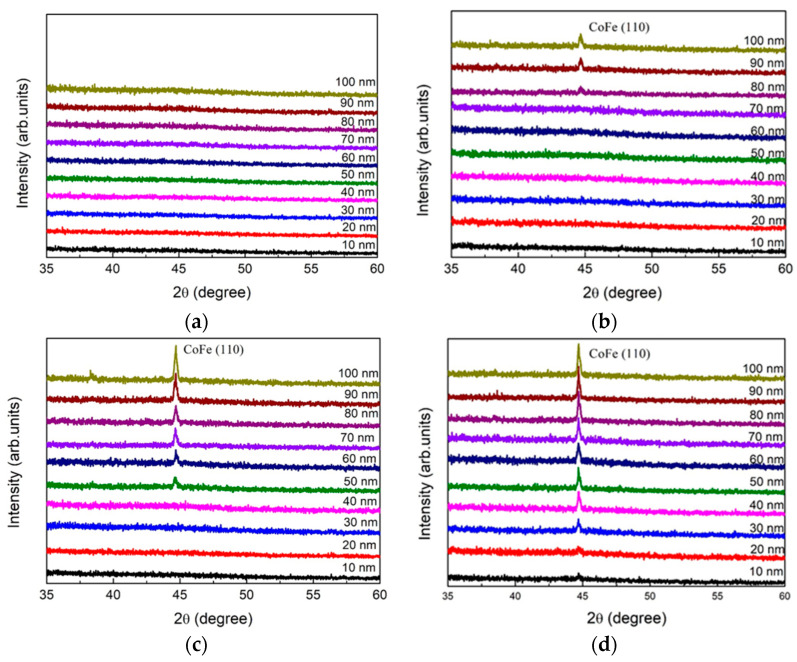

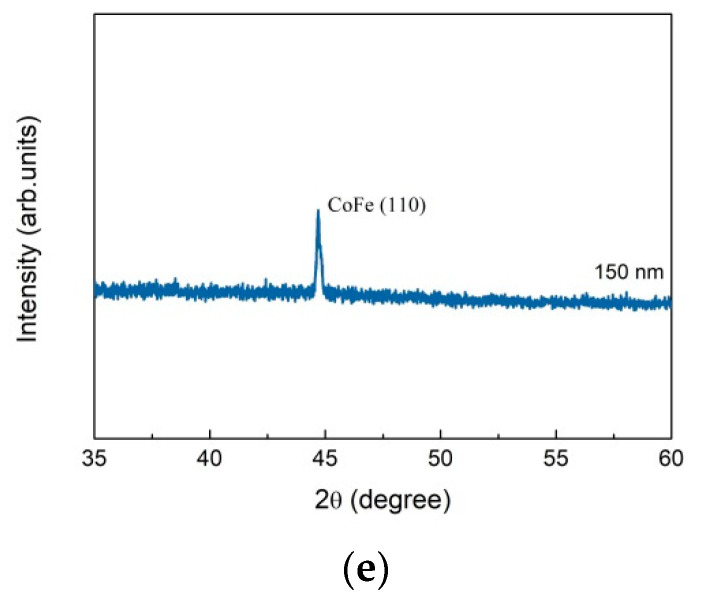
X-ray diffraction patterns of Co_40_Fe_40_W_10_B_10_thin films: (**a**) post-annealing at 200 °C, (**b**) post-annealing at 250 °C, (**c**) post-annealing at 300 °C, (**d**) post-annealing at 350 °C, and (**e**) post-annealing at 400 °C with 150 nm.

Figure 1a–e show Co_40_Fe_40_W_10_B_10_ thin films when analyzed for the crystal structure by XRD at the annealed temperatures of 200 °C, 250 °C, 300 °C, 350 °C, and 400 °C, respectively. Figure 1a of annealed 200 °C result displays an amorphous status in all films owing to insufficient heating drive force. Furthermore, the thicknesses and annealing temperatures of body-centered cubic (BCC) CoFe (110) peaks were detected at around a diffracted angle of 2θ = 44.7° when annealing at 250 °C with thicknesses from 80 nm to 100 nm, annealing at 300 °C with thicknesses from 50 nm to 100 nm, annealing at 350 °C with thicknesses from 10 nm to 100 nm, and annealing at 400 °C with thickness of 150 nm. It is generally observed in CoFeWB thin films that the intensity of CoFe(110) peaks increases with greater thickness and increased annealing temperature. When the annealing temperature increases above 350 °C, it is found that the thickness of the initial crystallization of CoFe (110) becomes thinner.

### 3.2. Full-Width at Half Maximum (FWHM) and Grain Size Distribution

**Figure 2:**
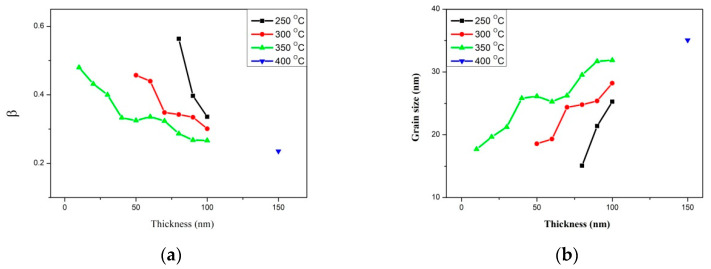
(**a**) Full-width at half maximum (FWHM) of CoFeWB thin films. (**b**) Grain size of CoFeWB thin films.

Figure 2a shows the corresponding full width at half maximum (FWHM, B) of the CoFe (110) peak obtained under four conditions. The result reveals that FWHM decreases with increased thicknesses and post-annealing temperature, using the FWHM determined by XRD, while the grain size of CoFe (110) is calculated using the Scherrer formula [21,22]. By using the Scherrer formula (1), and considering the CoFe (110) peak, this study calculated the average crystallite size for the CoFeWB thin films under the studied conditions.

The Scherrer formula is

D = Kλ/Bcosθ(1)

In the formula, k (0.89) is Scherrer’s constant; λ is the X-ray wavelength of the Cu Kα1 line; B is the FWHM diffraction CoFe (110) peak; and θ is the half-angle of the diffraction peak. Figure 2b shows the average grain sizes, which were estimated using the FWHM of the CoFe (110) peak under four annealed conditions. The results indicate that the grain sizes depend on the thickness and annealed temperature, and that the crystallization of the films rose with the thickness and annealed temperature, indicating annealing treatment supports the heating drive force to grain growth [23,24,25]. To investigate the thickness and annealing temperature effect, the performance of the grains at a thicker and higher temperature, 150 nm and 400 °C, was also studied. In Figure 2a, there is little difference in the FWHM and grain size when annealing at 350 °C with 100 nm and at 400 °C with 150 nm. The grain size of 400 °C is slightly larger than 350 °C. Therefore, the grain size tends to be saturated when annealing above 350 °C.

### 3.3. Magnetic Analysis

**Figure 3:**
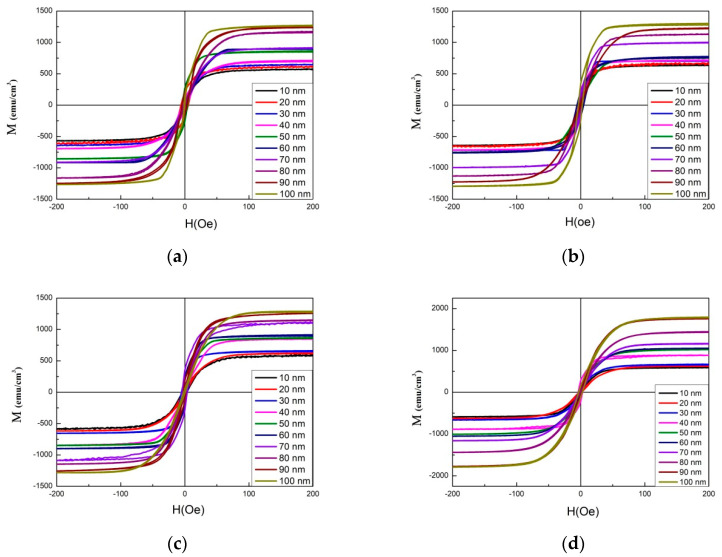
In-plane magnetic hysteresis loop of CoFeWB thin films: (**a**) after annealing at 200 °C, (**b**) after annealing at 250 °C, (**c**) after annealing at 300 °C, and (**d**) after annealing at 350 °C.

**Figure 4:**
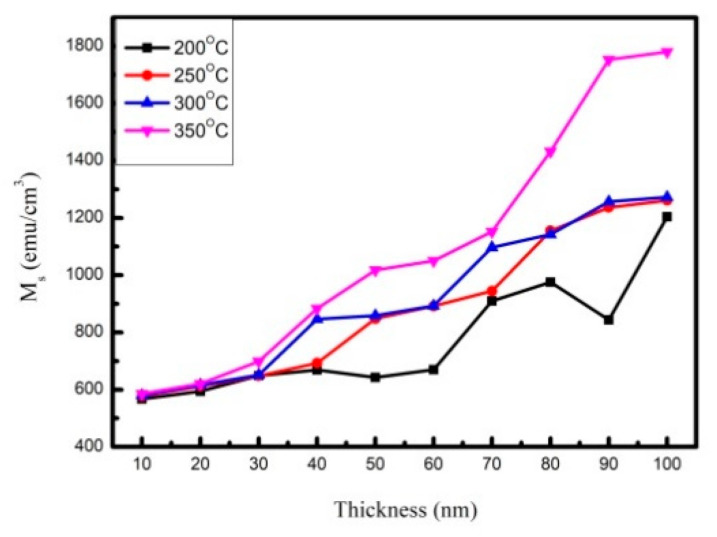
Saturation magnetization (M_S_) of CoFeWB thin films.

Figure 3a–d display the magnetic hysteresis loops of Co_40_Fe_40_W_10_B_10_ thin films under four annealed conditions, with thicknesses ranging from 10 to 100 nm. The external magnetic field of 200 Oe in the plane is enough to observe the saturation magnetic spin state. The figure shows low coercivity (H_C_), which indicates that Co_40_Fe_40_W_10_B_10_ films are soft magnetic. The saturation magnetization (M_S_) of Co_40_Fe_40_W_10_B_10_ thin films under four post-annealing conditions illustrates the magnetic properties of Co_40_Fe_40_W_10_B_10_ thin films that were measured by AGM, as shown in Figure 4. The results show that in Co_40_Fe_40_W_10_B_10_ films, Ms increases with the increase of thickness, indicating the effect of thickness on Ms. It was also found that the Ms of the CoFeWB thin films increased by raising the annealing temperature. Apparently, the M_S,_ when annealed at 350 °C, is much larger than other conditions because of magnetocrystalline anisotropy [26,27]. CoFeWB films show in-plane magnetization because the CoFeWB film is too thick and, when deposited on a Si substrate, perpendicular magnetic anisotropy (PMA) originates from the Fe-O bond and the in-plane demagnetization field is too big, owing to a thick CoFeWB [28,29]. In this study, the effect of greater CoFeWB thickness is demonstrated more than the weaker Fe-O bonding effect. The M_S_ value of Co_40_Fe_40_W_10_B_10_ thin films is increased to 350 °C, which shows that the thermal stability of Co_40_Fe_40_W_10_B_10_ thin films is better than that of other research findings [30].

**Figure 5:**
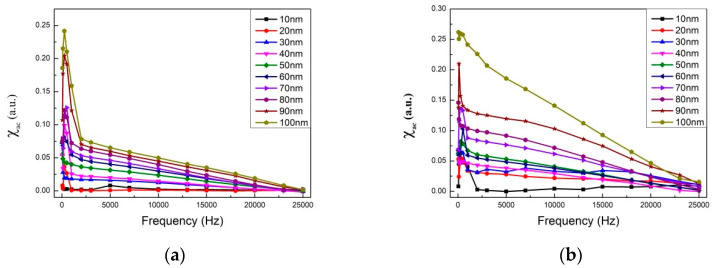

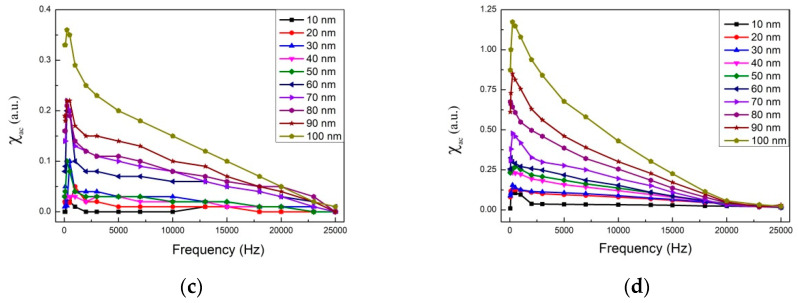
The low-frequency alternate-current magnetic susceptibility (χ_ac_) as a function of the frequency from 10 to 25,000 Hz: (**a**) after annealing at 200 °C, (**b**) after annealing at 250 °C, (**c**) after annealing at 300 °C, and (**d**) after annealing at 350 °C.

**Figure 6:**
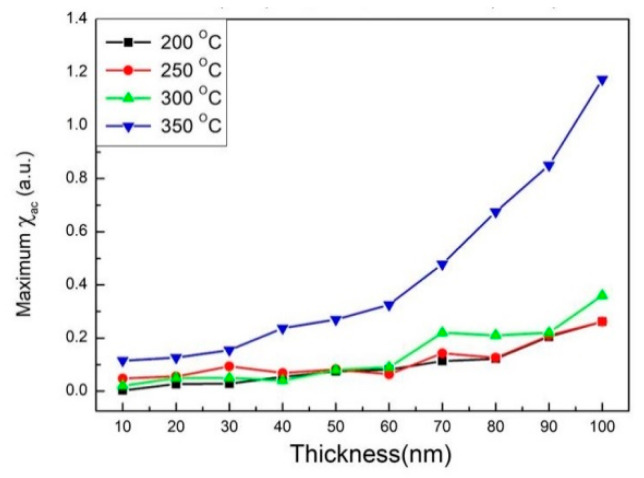
Maximum alternate-current magnetic susceptibility for the CoFeWB films.

Figure 5a–d show the low-frequency alternating-current magnetic susceptibility (χ_ac_) result of CoFeWB films with thicknesses ranging from 10 to 100 nm under four conditions. The low frequencies were measured in the range of 50–25,000 Hz. The results display that the χ_ac_ values decrease with an increasing frequency (Hz) under the four conditions. The corresponding maximum χ_ac_ values of various CoFeWB thicknesses under four conditions are shown in Figure 6. It was found that the maximum χ_ac_ value was 0.24 when the thickness was 100 nm at 200 °C; the maximum χ_ac_ value was 0.26 when the thickness was 100 nm at 250 °C; the maximum χ_ac_ value was 0.36 when the thickness was 100 nm at 300 °C; and the maximum χ_ac_ value was 1.17 when the thickness was 100 nm at 350 °C. These results clearly display an increased χ_ac_ owing to the thickness effect and magnetocrystalline anisotropy in the CoFeWB films. Table 1 shows the optimal resonance frequency (ƒ_res_) of CoFeWB. The maximum χ_ac_ demonstrates that the spin sensitivity is highest at the optimal resonant frequency [31]. The χ_ac_ peak indicates the spin exchange-coupling interaction and dipole moment of the domain under frequency. Additionally, the ƒ_res_ value is below 1000 Hz, which makes CoFeWB films for applications in soft magnetism devices. It was found that the ƒ_res_ values of all CoFeWB thicknesses were in the range of 50–1000 Hz, suggesting the maximum χ_ac_ had the strongest spin sensitivity at this frequency [32].

### 3.4. Contact Angle and Surface Energy

The contact angles were measured using DI water and glycerol. The data are presented in Table 2. The contact angles of all Co_40_Fe_40_W_10_B_10_ thin films were less than 90°, representing that Co_40_Fe_40_W_10_B_10_ film exhibits good hydrophilicity and wettability. The surface energy of thin films is an important parameter because it relates to the adhesion of thin films. When CoFeWB thin film is used as a seed, buffer, free, or pinned layer, the strong adhesion of the thin films is essential. The data of contact angles are used to calculate the surface energy via Young’s equation [19,20]:

σsg = σsl + σlg cosθ(2)

Here, σsg is the surface free energy of the solid; σsl denotes the liquid–solid interface tension; σlg is the surface tension of the liquid, and θ is the contact angle.

**Figure 7:**
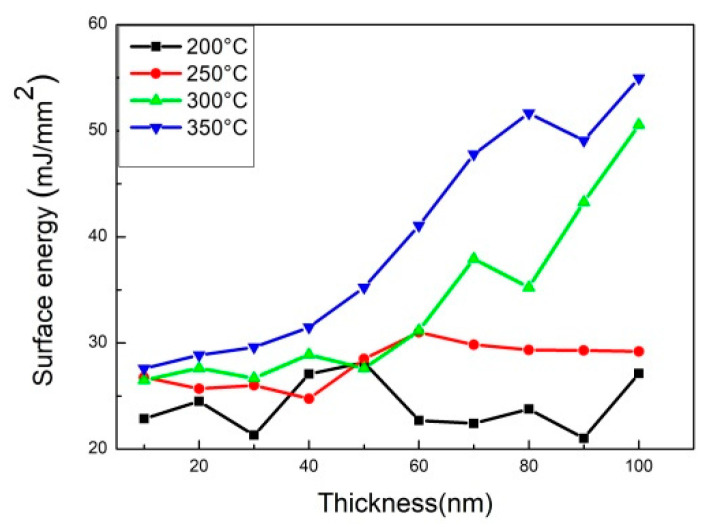
The surface energy of CoFeWB thin films.

Figure 7 displays the surface energy of Co_40_Fe_40_W_10_B_10_ thin films. These data of surface energy are shown in Table 2. It can be observed that the surface energy of high post-annealing temperature was larger than low annealing temperature. As the post-annealing temperature increased, the surface energy clearly increased. As a consequence, the surface energy of annealed 200 °C CoFeWB thin films was 28.15 mJ/mm^2^ at 50 nm, which was the highest value. When the post-annealing temperature was 250 °C, the highest surface energy of 60 nm was 31.01 mJ/mm^2^. When the post-annealing temperature achieved 300 °C, it was 50.56 mJ/mm^2^ at 100 nm. When the post-annealing temperature achieved 350 °C, it was 54.95 mJ/mm^2^ at 100 nm. When the surface energy is high, the liquid absorption capacity of the surface is correspondingly high. High surface energy corresponds to strong adhesion [33]. Surface energy is the key factor affecting the adhesion of the film. Because CoFeWB is compatible with spin-value MTJ and applied to MRAM application, it can also be used as a free layer and in combination with other layers. One can observe the Co_40_Fe_40_W_10_B_10_ properties and compare them with other specific Co_40_Fe_40_V_10_B_10_ film magnetic properties and surface energies, as mentioned in Table 3 [34]. The χ_ac_ and surface energy of 100 nm Co_40_Fe_40_W_10_B_10,_ annealed at 350 °C, is larger than that of the Co_40_Fe_40_V_10_B_10_ film, indicating that Co_40_Fe_40_W_10_B_10_ thin film is more suitable as a free or pinned layer in MTJ and can be applied in MRAM applications.

## 4. Conclusions

XRD patterns revealed that CoFeWB thin films are composed of an amorphous status when annealed at a temperature of 200 °C. BCC CoFe (110) peaks were detected when annealing at 250 °C with thicknesses ranging from 80 nm to 100 nm, annealing at 300 °C with thicknesses ranging from 50 nm to 100 nm, and annealing at 350 °C with thicknesses ranging from 10 nm to 100 nm. The intensity and grain size of CoFe (110) peaks generally increased with increasing film thickness and annealing temperatures, indicating a development in crystallographic texture. CoFeWB films presented soft magnetism owing to low H_c_ and in-plane magnetization because thicker CoFeWB thicknesses showed a large in-plane demagnetization field. M_s_ and χ_ac_ also increased as the thickness and annealed temperature increased. The χ_ac_ and M_S_ of films annealed at 350 °C are much larger than in other conditions, owing to the thickness effect and magnetocrystalline anisotropy in the CoFeWB films. Alloying additions of W improved the thermal stability of CoFeB films. The maximum Ms and χ_ac_ values were achieved for CoFeWB films with a thickness of 100 nm after annealing at 350 °C. The f_res_ values of all films were less than 1000 Hz. The contact angles of CoFeWB were smaller than 90°, indicating that the films were hydrophilic and had a good wetting effect. The surface energy of a high post-annealing temperature was larger than that of a low annealing temperature. As the post-annealing temperature increased, the surface energy clearly increased. Based on the magnetic properties and surface energy, the optimal condition of CoFeWB film is a thickness of 100 nm with an annealed temperature of 350 °C, which is suitable as a free or pinned layer in MTJ for magnetic component applications.

## Figures and Tables

**Table 1:** Optimal resonance frequency for films of various thicknesses.

Thickness (nm)	After Annealing at 200 °C (Hz)	After Annealing at 250 °C (Hz)	After Annealing at 300 °C (Hz)	After Annealing at 350 °C (Hz)
10	500	250	500	250
20	500	250	100	250
30	100	500	200	250
40	50	50	100	50
50	50	250	250	500
60	250	500	100	100
70	250	1000	250	250
80	250	50	250	50
90	250	100	500	250
100	250	50	250	250

**Table 2:** Comparing contact angle and surface energy for Co_40_Fe_40_W_10_B_10_ thin films from different fabrication processes.

Process	Thickness	Contact Angle with DI Water (θ)	Contact Angle with Glycerol (θ)	Surface Energy (mJ/mm^2^)
Post-annealing 200 °C	10 nm	90.0°	73.2°	22.87
	20 nm	80.7°	65.7°	24.50
	30 nm	85.8°	81.4°	21.31
	40 nm	84.3°	72.6°	27.09
	50 nm	80.5°	71.5°	28.15
Post-annealing 200 °C	60 nm	83.7°	79.4°	22.69
	70 nm	84.9°	84.0°	22.43
	80 nm	87.4°	81.7°	23.77
	90 nm	87.4°	83.0°	21.02
	100 nm	84.4°	72.7°	27.13
Post-annealing 250 °C	10 nm	82.5°	80.7°	26.77
	20 nm	85.6°	76.0°	25.69
	30 nm	86.5°	83.2°	26.01
	40 nm	80.7°	80.0°	24.75
	50 nm	84.2°	61.9°	28.49
Post-annealing 250 °C	60 nm	80.3°	77.3°	31.01
	70 nm	73.7°	70.0°	29.84
	80 nm	80.0°	71.4°	29.35
	90 nm	75.5°	70.6°	29.30
	100 nm	77.2°	75.2°	29.19
Post-annealing 300 °C	10 nm	82.2°	78.0°	26.49
	20 nm	77.2°	77.2°	27.62
	30 nm	80.5°	51.0°	26.68
	40 nm	77.8°	74.9°	28.90
	50 nm	83.4°	81.9°	27.61
Post-annealing 300 °C	60 nm	80.8°	78.2°	31.18
	70 nm	79.6°	65.9°	37.93
	80 nm	76.5°	72.4°	35.24
	90 nm	77.7°	76.6°	43.26
	100 nm	83.7°	73.0°	50.56
Post-annealing 350 °C	10 nm	78.3°	73.6°	27.60
	20 nm	75.6°	75.4°	28.85
	30 nm	76.9°	71.7°	29.61
	40 nm	71.6°	68.5°	31.48
	50 nm	81.1°	79.6°	35.24
Post-annealing 350 °C	60 nm	80.0°	78.0°	41.06
	70 nm	80.9°	77.2°	47.79
	80 nm	81.8°	79.7°	51.65
	90 nm	80.7°	73.9°	49.08
	100 nm	81.7°	75.6°	54.95

**Table 3:** Significant properties for Si(100)/Co_40_Fe_40_V_10_B_10_ and Si(100)/Co_40_Fe_40_W_10_B_10_ materials.

Material	Maximum χ_ac_ (a.u.)	Optimal Resonance Frequency, f_res_ (Hz)	Surface Energy (mJ/mm^2^)	Crystallinity
Si(100)/Co_40_Fe_40_V_10_B_10_ [34] 10–50 nm at RT	0.013–0.019	50–200	34.2–51.5	Weak
Si(100)/Co_40_Fe_40_W_10_B_10_ [*] 10–100 nm at annealed conditions[*]: Current research	0.24–1.17	50–1000	21.0–54.9	Strong

## Data Availability

Data sharing is not applicable.

## References

[1] Rozlin N.M.N., Alfantazi A.M. (2012). Nanocrystalline cobalt-iron alloy: Synthesis and characterization. Mater. Sci. Eng. A.

[2] Gibbs M.R.J., Hill E.W., Wright P.J. (2004). Magnetic materials for MEMS applications. J. Phys. D.

[3] Chen C., Xiong D., Gu M., Lu C., Yi F.Y., Ma X. (2020). MOF-Derived Bimetallic CoFe-PBA Composites as Highly Selective and Sensitive Electrochemical Sensors for Hydrogen Peroxide and Nonenzymatic Glucose in Human Serum. ACS Appl. Mater. Interfaces.

[4] Takemura Y., Inoue T., Nishimoto M., Yamada T. (2004). Fabrication of zero-speed sensor using weakly coupled NiFe/CoFe multilayer films. IEEE Trans. Magn..

[5] Takemura Y., Yamada T. (2006). Output properties of zero-speed sensors using FeCoV wire and NiFe/CoFe multilayer thin film. IEEE Sens. J..

[6] Zhang Z., Feng Y.C., Clinton T., Badran G., Yeh N.H., Tarnopolsky G., Girt E., Munteanu M., Harkness S., Richter H. (2002). Magnetic Recording Demonstration Over 100 Gb/in^2^. IEEE Trans. Magn..

[7] Li M., Shi H., Yu G., Lu J., Chen X., Han G., Yu G., Amiri P.K., Wang K.L. (2017). Effects of annealing on the magnetic properties and microstructures of Ta/Mo/CoFeB/MgO/Ta films. J. Alloys Compd..

[8] Almasi H., Hickey D.R., Newhouse-Illige T., Xu M., Rosales M.R., Nahar S., Held J.T., Mkhoyan K.A., Wang W.G. (2015). Enhanced tunneling magnetoresistance and perpendicular magnetic anisotropy in Mo/CoFeB/MgO magnetic tunnel junctions. Appl. Phys. Lett..

[9] Huang S.X., Chen T.Y., Chien C.L. (2008). Spin polarization of amorphous CoFeB detemined by point-contact Andreev reflection. Appl. Phys. Lett..

[10] Paluskar P.V., Attema J.J., de Wijs G.A., Fiddy S., Snoeck E., Kohlhepp J.T., Swagten H.J.M., de Groot R.A., Koopmans B. (2008). Spin tunneling in junctions with disordered ferromagnets. Phys. Rev. Lett..

[11] Li M., Wang S., Zhang S., Fang S., Yu G. (2019). The perpendicular magnetic anisotropies of CoFeB/MgO films with Nb buffer layers. J. Magn. Magn. Mater..

[12] Meng H., Lum W.H., Sbiaa R., Lua S.Y.H., Tan H.K. (2011). Annealing effects on CoFeB-MgO magnetic tunnel junctions with perpendicular anisotropy. J. Appl. Phys..

[13] Pai C.F., Liu L., Li Y., Tseng H.W., Ralph D.C., Buhrman R.A. (2012). Spin transfer torque devices utilizing the giant spin Hall effect of tungsten. Appl. Phys. Lett..

[14] An G.G., Lee J.B., Yang S.M., Kim J.H., Chung W.S., Hong J.P. (2015). Highly stable perpendicular magnetic anisotropies of CoFeB/MgO frames employing W buffer and capping layers. Acta Mater..

[15] Ghaferi Z., Sharafi S., Bahrololoom M.E. (2016). The role of electrolyte pH on phase evolution and magnetic properties of CoFeW codeposited films. Appl. Surf. Sci..

[16] Liu Y., Yu T., Zhu Z., Zhong H., Khamis K.M., Zhu K. (2016). High thermal stability in W/MgO/CoFeB/W/CoFeB/W stacks via ultrathin W insertion with perpendicular magnetic anisotropy. J. Magn. Magn. Mater..

[17] Wang H., Kou X., Wang S., Zhou J., Zhang X., Li J. (2011). Structures, magnetic properties and thermal stability of CoFeB/MgO films. Phys. Procedia.

[18] Ma K., Chung T.S., Good R.J. (1998). Surface energy of thermotropic liquid crystalline polyesters and polyesteramide. J. Polym. Sci..

[19] Owens D.K., Wendt R.C. (1969). Estimation of the surface free energy of polymers. J. Appl. Polym. Sci..

[20] Kaelble D.H., Uy K.C. (1970). A Reinterpretation of Organic Liquid-Polytetrafluoroethylene Surface Interactions. J. Adhens..

[21] Cullity B.D. (1978). Elements of X-ray Diffraction.

[22] Patterson A.L. (1939). The Scherrer formula for X-Ray particle size determination. Phys. Rev..

[23] Jin Y., Lin B., Bernacki M., Rohrer G.S., Rollett A.D., Bozzolo N. (2014). Annealing twin development during recrystallization and grain growth in pure nickel. Mater. Sci. Eng. A.

[24] Lu Y., Duan F., Pan J., Li Y. (2021). High-throughput screening of critical size of grain growth in gradient structured nickel. J. Mater. Sci. Technol..

[25] Mouhib F.Z., Sheng F., Mandia R., Pei R., Korte-Kerzel S. (2021). Texture selection mechanisms during recrystallization and grain growth of a magnesium-erbium-zinc alloy. Metals.

[26] Budde T., Gatzen H.H. (2004). Magnetic properties of an SmCo/NiFe system for magnetic microactuators. J. Magn. Magn. Mater..

[27] Wen D., Li J., Gan G., Yang Y., Zhang H., Liu Y. (2019). Double peaks of the permeability spectra of obliquely sputtered CoFeB amorphous films. Mater. Res. Bull..

[28] Iihama S., Mizukami S., Naganuma H., Oogane M., Ando Y., Miyazaki T. (2014). Gilbert damping constants of Ta/CoFeB/MgO(Ta) thin films measured by optical detection of precessional magnetization dynamics. Phys. Rev. B.

[29] Li M., Wang S., Zhang S., Fang S., Feng G., Cao X., Zhang P., Wang B., Yu G. (2019). The effect of interfacial oxygen migration on the PMA and thermal stability in MTJ with double MgO layers. Appl. Sur. Sci..

[30] Miyakawa N., Worledge D.C., Kita K. (2013). Impact of Ta diffusion on the perpendicular magnetic anisotropy of Ta/CoFeB/MgO. IEEE Magn. Lett..

[31] Yang S.Y., Chien J.J., Wang W.C., Yu C.Y., Hing N.S., Hong H.E., Hong C.Y., Yang H.C., Chang C.F., Lin H.Y. (2011). Magnetic nanoparticles for high-sensitivity detection on nucleic acids via superconducting-quantum-interference-device-based immunomagnetic reduction assay. J. Magn. Magn. Mater..

[32] Chen Y.T., Xie S.M., Jheng H.Y. (2013). The low-frequency alternative-current magnetic susceptibility and electrical properties of Si(100)/Fe_40_Pd_40_B_20_(X Å)/ZnO(500 Å) and Si(100)/ZnO(500 Å)/Fe_40_Pd_40_B_20_(Y Å) systems. J. Appl. Phys..

[33] Porter D.A., Easterling K.E. (1992). Phase Transformations in Metals and Alloy.

[34] Ou S.L., Liu W.J., Chang Y.H., Chen Y.T., Wang Y.T., Li W.S., Tseng J.Y., Wu T.H., Chi P.W., Chu C.L. (2020). Structure, magnetic property, surface morphology, and surface energy of Co_40_Fe_40_V_10_B_10_ films on Si(100) substrate. Appl. Sci..

